# An extremely low stomatal density mutant overcomes cooling limitations at supra-optimal temperature by adjusting stomatal size and leaf thickness

**DOI:** 10.3389/fpls.2022.919299

**Published:** 2022-07-22

**Authors:** María Luisa Pérez-Bueno, Jonatan Illescas-Miranda, Amanda F. Martín-Forero, Alberto de Marcos, Matilde Barón, Carmen Fenoll, Montaña Mena

**Affiliations:** ^1^Facultad de Ciencias Ambientales y Bioquímica, Universidad de Castilla-La Mancha, Toledo, Spain; ^2^Departamento de Bioquímica, Biología Celular y Molecular de Plantas, Estación Experimental del Zaidín, Consejo Superior de Investigaciones Científicas (CSIC), Granada, Spain; ^3^Departamento de Fisiología Vegetal, Universidad de Granada, Granada, Spain

**Keywords:** supra-optimal temperature, stomatal development, photosynthesis, transpiration, heat adaptation, thermomorphogenesis, autofluorescence imaging

## Abstract

The impact of global warming on transpiration and photosynthesis would compromise plant fitness, impacting on crop yields and ecosystem functioning. In this frame, we explored the performance of a set of Arabidopsis mutants carrying partial or total loss-of-function alleles of stomatal development genes and displaying distinct stomatal abundances. Using microscopy and non-invasive imaging techniques on this genotype collection, we examined anatomical leaf and stomatal traits, plant growth and development, and physiological performance at optimal (22°C) and supra-optimal (30°C) temperatures. All genotypes showed thermomorphogenetic responses but no signs of heat stress. Data analysis singled out an extremely low stomatal abundance mutant, *spch-5*. At 22°C, *spch-5* had lower transpiration and warmer leaves than the wild type. However, at 30°C, this mutant developed larger stomata and thinner leaves, paralleled by a notable cooling capacity, similar to that of the wild type. Despite their low stomatal density (SD), *spch-5* plants grown at 30°C showed no photosynthesis or growth penalties. The behavior of *spch-5* at supra-optimal temperature exemplifies how the effect of very low stomatal numbers can be counteracted by a combination of larger stomata and thinner leaves. Furthermore, it provides a novel strategy for coping with high growth temperatures.

## Introduction

Global warming has already imposed previously unseen constrictions to plant growth and reproduction (IPCC, 2019). Current models predict temperature increases that, even in the most optimistic scenarios, will endanger productivity of wild and crop plants, as in some geographical areas will concur with water shortages during the growing and reproductive seasons of many species (IPCC, 2014; Zhao et al., 2017). These moderate but sustained increases of a few degrees over the optimum growth temperature for a species are referred to as supra-optimal temperatures, and they influence plant growth and development in ways different from heat stress, as reviewed by Casal and Balasubramanian (2019). The adaptive changes that plants experience during growth at supra-optimal temperature are collected under the term thermomorphogenesis and affect a number of anatomical, morphological, phenological, and physiological traits whose molecular and genetic bases are only beginning to be unraveled (Casal and Balasubramanian, 2019; Lippmann et al., 2019; Vu et al., 2019; Ding et al., 2020). In Arabidopsis (*Arabidopsis thaliana*) seedlings, PHYTOCHROME-INTERACTING FACTOR 4 (PIF4) acts as a central hub where other signaling pathways involving light, hormones, and circadian clocks, as well as epigenetic processes related to chromatin remodeling converge to modulate the morphogenetic responses to supra-optimal temperature (reviewed in Casal and Balasubramanian, 2019; Vu et al., 2019; Qiu, 2020).

Some of the Arabidopsis responses to growth at supra-optimal temperature, such as elongated leaf petioles or erect (thermonasty) and thinner leaves, contribute to promote cooling, what plants do mostly through transpiration (Crawford et al., 2012). Transpiration takes place through stomata, the dynamic valves in the epidermis of aerial organs and through which most of the gas exchange between the plant and the atmosphere takes place. As stomata regulate water loss but also CO_2_ uptake, they are key structures for photosynthesis as well. Stomata open or close through shape changes in the two guard cells that delimit the stomatal pore, and do so in response to ambient conditions and internal cues, thus regulating the surface available for gas exchange (Meng et al., 2015; Qi and Torii, 2018). When stomata open, H_2_O vapor loss to the atmosphere not only produces the evaporative cooling of the surface but also the transpiration current that promotes water and nutrient uptake at the soil-root interphase. Stomatal dynamics is finely tuned to maintain equilibrium between water loss and CO_2_ uptake under different environmental and physiological scenarios (Grill and Ziegler, 1998). The modification of key regulators of stomata movements may render surprising results. For instance, tomato plants showed increased growth without extra water loss under fluctuating light by the introduction of an artificial light-activated K^+^ channel that made stomata open faster during sun flecks (Papanatsiou et al., 2019). This work is one of many examples highlighting how stomata-related processes can affect growth and water use efficiency.

In addition to stomatal movements, anatomical aspects linked to stomata are emerging as crucial to plant performance under changing environments. The maximum area available for gas exchange between the leaf and the atmosphere (the theoretical maximum stomatal conductance, *g*_smax_) depends on the number, size, and spatial distribution of the stomata on the leaf surfaces (Dow and Bergmann, 2014; Lawson and Blatt, 2014; Franks et al., 2015; Harrison et al., 2020). Controlled cell division and differentiation events during organ development determine these traits. This takes place through a complex network of molecular regulators that are genetically determined and influenced by environmental cues (Hetherington and Woodward, 2003; Pillitteri and Dong, 2013; Qi and Torii, 2018; Bertolino et al., 2019; Han et al., 2021). Arabidopsis has served as a model species to dissect such circuits, resulting in the identification of a number of genes that determine not just the formation but also the abundance and spatial distribution of stomata (Nadeau and Sack, 2002; Bergmann and Sack, 2007; Pillitteri and Torii, 2012; Zoulias et al., 2018; Herrmann and Torii, 2021). Many of such genes encode negative regulators that restrict stomatal fate, their loss of function rendering excessive stomata. This is the case of the apoplastic signaling peptides EPIDERMAL PATTERNING FACTORS 1 and 2 (EPF1 and EPF2; Torii, 2015). These peptides are perceived by membrane receptor complexes that contain combinations of receptor-like kinases, notably ERECTA and ERECTA-LIKE 1 and 2 (ER, ERL1, and ERL2), as well as TOO MANY MOUTHS (TMM), a receptor-like protein that lacks the kinase domain (Meng et al., 2015; Han and Torii, 2016; Ho et al., 2016; Lee and Bergmann, 2019). Mutants in any of these peptides and receptors or their combinations, which are specific to different cell types in the developing stomatal lineages, produce more stomata that are often clustered. Upon peptide binding, the receptors signal a mitogen-activated protein kinase (MAPK) phosphorylation cascade led by the MAPKKK YODA, resulting in the inactivation of a set of transcription factors that are the main positive drivers of stomatal development (Pillitteri and Torii, 2007; Lampard et al., 2008, 2009). Among them is SPEECHLESS (SPCH), responsible for the initiation of stomatal lineages (MacAlister et al., 2007). A lack of function of these key positive factors renders stomata-less plants and is seedling lethal (Pillitteri and Dong, 2013; de Marcos et al., 2015). However, their hypomorphic alleles such as *spch-5* allow stomata to be made, although in reduced numbers, producing viable and fertile plants (de Marcos et al., 2017). Interestingly, among the thermomorphogenic responses described in Arabidopsis is a reduction in stomatal abundance (Crawford et al., 2012; Vile et al., 2012) based on the interaction of PIF4, with the master inducer of stomatal development SPEECHLESS (Lau et al., 2018).

Several studies have exploited mutant or transgenic Arabidopsis lines with modified activity of positive and negative stomatal development regulators to interrogate the physiological consequences of their altered stomatal abundance under several growth conditions (Masle et al., 2005; Tanaka et al., 2013; Dow et al., 2014; Franks et al., 2015; Vráblová et al., 2017; Zoulias et al., 2018). These and other studies indicate that the correlations between anatomical parameters and stomatal conductance are far from simple, and no univocal links have been found (reviewed by Bertolino et al., 2019; Endo and Torii, 2019; Harrison et al., 2020). For instance, although it seems logical that a lower stomatal density (SD) should reduce transpiration and photosynthesis, while higher stomata densities would increase both parameters, this is not always the case, and different authors report different results (Doheny-Adams et al., 2012; Dow et al., 2014; Frank et al., 2015; Hepworth et al., 2015, 2016; Wang et al., 2016). The current view is that environmental factors set the different responses that relate changes in anatomical stomatal traits that influence theoretical maximal (*g*_smax_) and actual (*g*_s_) stomatal conductance with physiological processes, such as photosynthesis and transpiration (Frank et al., 2015; Harrison et al., 2020). Crawford et al. (2012) reported that well-watered Arabidopsis plants developed at supra-optimal temperature showed a decreased SD but sustained an increased transpiration, indicating that adaptations to high temperature may enhance evaporative leaf cooling, at least when water was available.

Therefore, there is a need for more experimental data that examine the performance of genotypes exhibiting different stomatal phenotypes, and under different environmental conditions, particularly those that will prevail in the future climate. In this work, we analyzed, at optimal (22°C) and supra-optimal (30°C) growth temperatures, a set of Arabidopsis mutants carrying hypomorphic or severe alleles for stomatal development genes, which display distinct stomatal abundance phenotypes. We examined (i) anatomical traits, such as SD, stomata and stomatal pore size, and leaf thickness and structure; (ii) plant growth by measuring bolting time, leaf numbers, the projected rosette area and dry weight (DW) in the various genotypes grown under the two conditions; (iii) physiological performance of the mutants in the two conditions through non-invasive imaging techniques, including their photosynthetic activity and their leaf temperature, as an estimation of transpiration. All genotypes showed the thermomorphogenetic responses described for Arabidopsis, albeit to different extents, and no signs of heat stress. Through this analysis, we singled out an extremely low stomatal abundance mutant previously isolated in our laboratory, *spch-5.* We found that, at supra-optimal temperature, *spch-5* underwent significant anatomical changes, paralleled by a notable cooling capacity with no photosynthesis or growth penalties. Our study provides further support to the notion that plants unfold a wide arrangement of different strategies aimed at coping with high growth temperatures.

## Materials and methods

### Plant material and growth conditions

Seeds of *A. thaliana* (L.) Heynh., accession Columbia-0 (Col-0), *epf1-1* (Hara et al., 2007), *epf2-3* (Hara et al., 2009), *er-105* (Torii et al., 1996), and *er-116* (Lease et al., 2001) were obtained from the Nottingham *Arabidopsis* Stock Centre; we have described previously the mutants *spch-5* (de Marcos et al., 2017) and *sdd1-3* (Delgado et al., 2012)*; tmm-1* and *spch-2* were kind gifts of Fred Sack (The University of British Columbia; Nadeau and Sack, 2002), and Dominique Bergmann (Stanford University; MacAlister et al., 2007), respectively. All mutant lines were in Col-0 background and have an SD phenotype. Some of them also show a substantial proportion of clustered stomata. The *er-116* stomatal phenotype is firstly described in this study.

The seeds were germinated under optimal conditions, as follows: 22/18°C day/night temperature, a 16-h photoperiod, 120 μE m^–2^s^–1^, 60% relative humidity. Typically, radicle emergence took place at 3 days after sowing, and the plants were subsequently grown under the same conditions. For experiments under supra-optimal temperature, the seeds were also germinated under optimal conditions and seedlings transferred to 30/24°C day/night 3 days after germination (dag) to avoid potential selection bias by temperature during seedling establishment. For short-term supra-optimal treatments, the plants were grown under optimal conditions for 15 dag and moved to 30/24°C day/night for the last 3 days prior to measurements. All the plants were cultivated in growth chambers, on pots containing soil Compo Sana (Compo, Spain), and watered three times a week.

### Plant physiology

#### Photosynthetic activity and secondary metabolism

The activity of the electron transport chain of the chloroplast was analyzed by chlorophyll fluorescence kinetics using a customized kinetic imaging fluorometer Open FluorCam FC 800-O (PSI, Brno, Czechia), as previously described (Granum et al., 2015). Briefly, red fluorescence was registered on plants after 30-min dark adaptation before (F_0_) and after (Fm) a 1-s saturating light pulse of 2,000 μE m^–2^ s^–1^. Subsequently, the plants were light adapted to 450 μE m^–2^ s^–1^ light for 10 min. During this light adaptation, saturating light pulses were provided at set times in order to record Ft and Fm’ before and after each light pulse, respectively. PSII maximum efficiency (Fv/Fm, in the dark-adapted state) and effective quantum efficiency of PSII [Φ_PSII_, (Fm′-Ft)/Fm′] and non-photochemical quenching (NPQ) of absorbed light in PSII [NPQ, (Fm-Fm′)/Fm′] during the light adaptation and at steady state were calculated according to Maxwell and Johnson (2000). Fluorescence kinetics was recorded at the corresponding growing temperature. The accumulation of secondary metabolites was analyzed by multicolor fluorescence imaging using the previously mentioned Open FluorCam FC 800-O. Blue and green fluorescence emitted by leaves excited by UV light was recorded and analyzed according to Granum et al. (2015). All measurements were performed 15 dag.

#### Thermography

Thermal images of the whole plants were recorded using a FLIR A305sc camera (FLIR Systems, Wilsonville, OR, United States) vertically positioned 30 cm above the plants, according to Pérez-Bueno et al. (2016). For each measurement, 10 thermal images were collected over 10 s in the growth chamber and always at midday. These images were used to generate an image of average values from where average temperature values were extracted for each cotyledon and fully expanded leaf individually. Image processing was carried out using the FLIR Research and Development software. Measurements were performed 15 dag.

#### Hyperspectral reflectance

Reflectance spectra in the 400- to 1,000-nm range of plant rosettes were collected using a Pika L hyperspectral imaging camera (Resonon, Bozeman, MT, United States). The obtained spectra were used to calculate vegetation indexes (VIs) and other reflectance parameters on fully developed leaves. The parameters taken in consideration are related to different aspects of plant fitness, leaf composition, and structure (Gitelson et al., 1996; Behmann et al., 2015; Roberts et al., 2018) and are the following: physiology (a green normalized difference vegetation index, GNDVI; and a photochemical reflectance index, PRI); stress (a red-edge vegetation stress index, RVSI); pigments (a pigment-specific spectral ratio, PSSR2); content on chlorophylls (a chlorophyll absorption reflectance index, CARI), anthocyanins (an anthocyanin reflectance index, ANTH2), and carotenes (a carotenoid reflectance index, CAR2); and structure (a modified red-edge normalized difference vegetation index, mRENVDI). Measurements were performed at 15 dag.

### Plant growth and morphology

Plant growth was analyzed in terms of morphology and plant biomass. Measurements were performed at 15 dag, with the exception of DW at bolting (DW_*bolting*_), which was determined at 23 dag for plants grown at 22°C, and at 18 dag for those grown at 30°C. The number of fully developed leaves was registered for each plant. For the determination of the projected plant area, the plants were scanned in a standard office scanner and the images were analyzed with ImageJ 1.49v (NIH, Bethesda, MS, United States). DW of whole rosettes was measured after drying in an oven at 60°C for 2 days.

### Microscopy and analysis of epidermal phenotypes and leaf thickness

The abaxial cotyledon and the leaf abaxial and adaxial epidermis were examined by differential interference contrast (DIC) microscopy in fixed fully expanded organs (23 dag or 17 dag at optimal and supra-optimal growth temperature, respectively), as described in Delgado et al. (2011) and Triviño et al. (2013), with minor modifications. Organs were hand excised, fixed for 16 h in ethanol:acetic acid 9:1 (v/v), and then replaced by 90% (v/v) ethanol, and rehydrated by serial 1-h incubations in ethanol:water (v/v) of decreasing ethanol content (70, 50, 30, and 10%), with a final step of distilled water, at room temperature. The specimens were then clarified in a chloral hydrate:glycerol:water solution (8:1:2, w/v/v) and observed under a Nikon Eclipse 90i upright microscope with DIC optics and photographed with a DXM1200C camera.

Stomatal Density (SD; number of stomata/mm^–2^) was calculated in the fixed organs by counting stomata in two areas of 0.4 mm^2^ located on both sides of the median axis of the cotyledon or leaf, modified from Delgado et al. (2011). Clusters were scored as groups of two or more stomata in direct contact. SD was calculated from counts of 7 plants (*n* = 7). Stomatal size as previously defined (Franks and Beerling, 2009) was calculated in μm^2^ as guard cell length multiplied by guard cell pair width. The stomatal pore length (*p*) was measured as the length of the pore at the longest axis (μm). The maximum stomatal pore area (*a*_max_) was defined as a circle with the diameter equal to *p* (*a*_max_ = π *p*^2^/4) and expressed in μm^2^. Stomatal pore depth (*l*) was estimated by single guard cell width. All measurements were made in micrographs of fixed organs using ImageJ (Schneider et al., 2012), and stomatal parameters were obtained for each side of the leaf by scoring 100 stomata from 7 plants.

The measured anatomical parameters were used to calculate the maximum stomatal conductance (*g*_smax_) as the potential area available for gas exchange with the following equation (Franks and Farquhar, 2001; Franks and Beerling, 2009):


$$g_{\text{smax}}=\frac{d}{v}\cdot D\cdot a_{\text{max}}/\left(l+\frac{\pi }{2}\cdot \sqrt{a_{\text{max}}/\pi }\right)$$

where *d* is the diffusivity of water vapor in air and *V* the molar volume of air at the growth temperatures (22 and 30°C), *D* is the SD value determined for each leaf, while *l* (the stomatal pore depth) and *a*_max_ were the genotype average values. For each plant, the adaxial and abaxial *g*_smax_ were calculated and used to obtain the total for the leaf (*g*_smaxT_ = adaxial *g*_smax_ + abaxial *g*_smax_).

Leaf thickness was measured on micrographs of fully expanded leaf transections. Leaf samples of approximately 4 mm^2^ were cut with a razor blade and immediately fixed in 1% paraformaldehide, 2% glutaraldehide, and 50-mM cacodilate (pH = 7.4) for 30 min under a vacuum. The samples were post-fixed in 1% OsO_4_ with 1% K_3_Fe(CN)_6_ for 1 h. The samples were dehydrated in subsequent EtOH solutions (50, 70, 90, and 100% in water). The samples were finally embedded in EMbed 812 (Electron Microscopy Sciences, Hatfield, PA, United States). Semithin 0.5-μm transections were obtained with ultramicrotome Reichert Jung S (Leica Microsystems, Wetzlar, Germany) and stained in 1% toluidine blue O and 1% borax. Sections were observed under an inverted microscope Leica DMi6000 (Leica Microsystems) and images analyzed by Adobe Photoshop (Adobe Systems Incorporated, San Jose, CA, United States). The average leaf thickness was calculated from leaf volume to area ratio (LVA) measured on micrographs as the volume corresponding to a 200-μm wide region of a 1-μm thick section, according to Poorter et al. (2009). For selected genotypes, the percentage of the total area corresponding to spongy and palisade mesophyll, and the adaxial and abaxial epidermis was determined in 200-μm wide micrograph regions. Fully expanded leaves were collected at 23 dag for optimal, and at 17 dag for supra-optimal growth temperatures.

### Statistical analysis

Unless otherwise specified, statistical analyses were performed using the SPSS version 28.0 software (IBM Corporation, Armonk, NY, United States). All experiments were carried out three times, providing comparable results. The data shown were from a representative experiment. Normality of the data and homogeneity of variance were verified by the Shapiro–Wilk and Levene tests, respectively. Student’s *t*-test was used for comparing two means. For multiple comparisons, the data were analyzed by one-way ANOVA followed by either the Tukey’s or Dunnett’s T3 *post hoc* test, or the Kruskal–Wallis test followed by the Dunn’s test. Principal components analysis (PCA) and hierarchical clustering on principal components (HCPC) were carried out on normalized and standardized variables using RStudio version 1.0.136 (RStudio, Inc., Boston, MA, United States). The parameters considered for PCA and subsequent HCPC were selected following the criteria of no redundancy (to avoid overfeeding the PCA) and maximum-explained variance.

### Parameter symbols and descriptions

The symbols and descriptions of the parameters used in this study are listed in Supplementary Table 1.

## Results

### Physiological traits of stomatal abundance and pattern mutants

**FIGURE 1:**
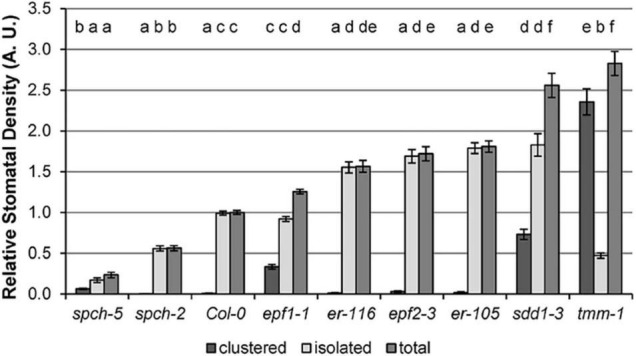
Stomatal density in the abaxial cotyledon epidermis of Col-0 and stomata developmental mutants. Values are shown relative to Col-0, and error bars represent ± SE of the means. Incidence of single, clustered, and total stomata is depicted. Different letters above the bars indicate statistically significant differences between genotypes (*p* ≤ 0.05) according to one-way ANOVA, followed by the Dunnet’s T3 test (isolated and total stomata data) or the Kruskal–Wallis test followed by the Dunn’s test (clustered stomata data). Data were obtained from fully expanded cotyledons (23 dag) of 10 plants (*n* = 10).

To evaluate the impact of SD on physiological traits under optimal growth temperature (22°C), we first selected eight mutant genotypes, displaying a range of SD values, using Col-0 as the wild-type control. In cotyledons, they encompass a broad SD variation, ranging from about 20–280% of the SD values in Col-0 (Figure 1). Some of the mutant phenotypes concurred with moderate to severe stomatal clustering. Both traits are shown in absolute values (Supplementary Figure 1) and relative to Col-0 values (Figure 1). Stomatal Index, a parameter that measures the proportion of epidermal cells that are stomata, was not considered, as it is not directly related to the gas exchange potential.

**FIGURE 2:**
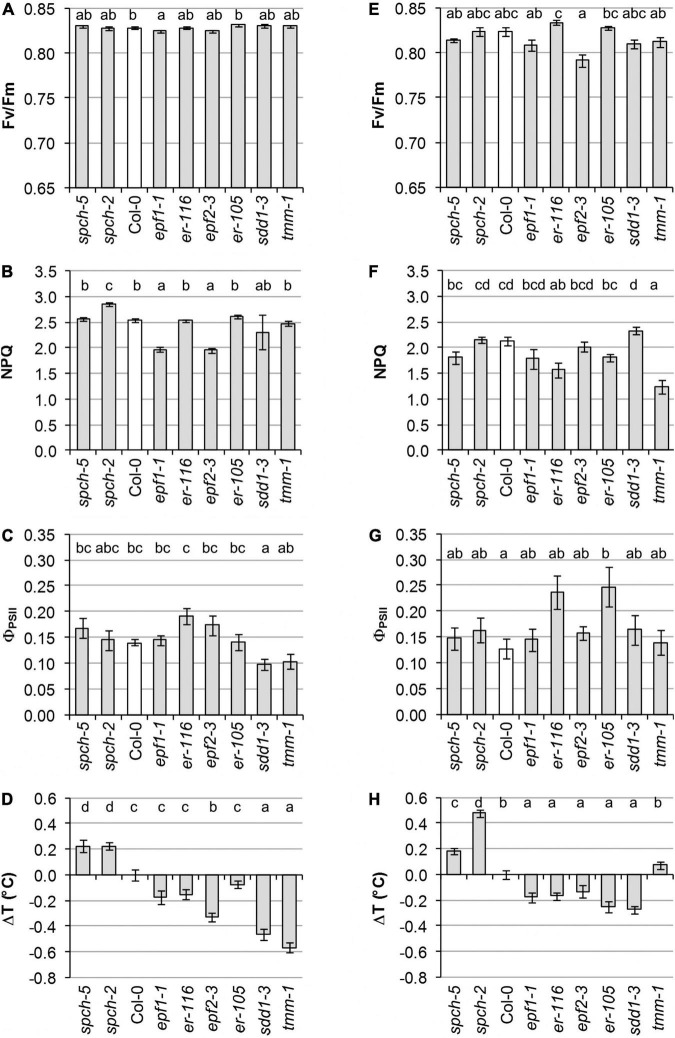
Physiological performance of different stomatal abundance genotypes. Measurements were performed in cotyledons at 8 dag **(A–D)** and leaves at 23 dag **(E–H)**. **(A,E)** Photosynthetic capacity is evaluated in terms of maximal efficiency of PSII (F_*V*_/F_*M*_). **(B,F)** The capacity for energy dissipation in the chloroplast is analyzed by the non-photochemical quenching in steady state (NPQ). **(C,G)** Photosynthetic activity is evaluated as quantum yield of PSII in steady state (Φ_PSII_). **(D,H)** Alteration in temperature of cotyledons and leaves of mutant lines relative to the wild type. Values are means ± SE, calculated from *n* = 20 **(A–D,H)** and *n* = 12 **(E–G)**. Different letters above the bars indicate statistically significant differences between genotypes at the same growth temperature (*p* ≤ 0.05) according to the Kruskal–Wallis test followed by the Dunn’s test **(A–E,H)** or one-way ANOVA followed by the Tukey’s test **(F,G)**.

Cotyledons and first leaves were examined for physiological performance through non-invasive imaging techniques to determine differences in the photosynthetic activity by means of PSII functionality (by F_*V*_/F_*M*_), PSII activity (by Φ_PSII_), and capacity for energy dissipation in the PSII (by NPQ) (Figure 2 and Supplementary Figure 2). The organ temperature was also recorded to estimate differences in transpiration. Compared to Col-0, cotyledons of the *epf* mutants were affected in PSII behavior, showing a decrease in the capacity for energy dissipation (NPQ, Figure 2B and Supplementary Figure 2B) that, however, did not cause a significant inhibition of PSII activity (Figure 2C). A slight photoinhibition of PSII functionality was also observed in *epf1-1* cotyledons (Figure 2A). In contrast, the two *er* mutations had effects on leaves, with *er-116* decreasing energy dissipation as NPQ (Figure 2F) and only *er-105* showing greater PSII activity values than those of Col-0 (Figure 2G). The *sdd1-3* and *tmm-1* stomata-clustering mutants exhibited different organ effects, with *sdd1-3* showing reduced PSII activity in cotyledons (Figure 2C) and *tmm-1* decreased energy dissipation in leaves (Figure 2F). Remarkably, despite their low SD, the *spch* mutants showed wild-type values for all PSII parameters in both organs, except for an increase in NPQ in *spch-2* cotyledons (Figure 2B). NPQ measures energy loss as heat and fluorescence, but the contribution of the heat dissipated to leaf temperature is negligible compared to transpiration (Jones, 1999).

Leaf temperature mostly agreed with the SD values characteristic of the various genotypes, yet only some mutations impacted on cotyledon temperature (Figures 2D,H). In general, mutants with increased SD had colder leaves than Col-0, whereas the low SD *spch* mutants were significantly warmer. Deviating from this tendency, *tmm-1* leaves showed the same temperature than those of Col-0. Consistent with the behavior of leaves, cotyledons of the *spch* mutants displayed the highest temperature values. The *sdd1*-*3* and *tmm*-*1* mutations, both causing high SD increases, led to much colder cotyledons than Col-0. A lower temperature was observed in *epf2*-*3* cotyledons, while the rest of the mutants with moderate SD increases showed no significant differences with respect to Col-0.

### Impacts of supra-optimal growth temperature

In order to assess the possible effects of SD alterations on plant growth and physiology, further experiments were carried out on a selection of genotypes in which the plants were grown at supra-optimal temperature (30°C). From the initial panel, genotypes with no or mild alterations in photosynthetic parameters were chosen: *er-116*, with increased SD; *tmm-1*, with increased SD but presenting a large fraction of clustered stomata; plus *spch-2* and *spch-5*, with SD decreased to a different extent.

#### Effects of supra-optimal temperature on plant growth

**FIGURE 3:**
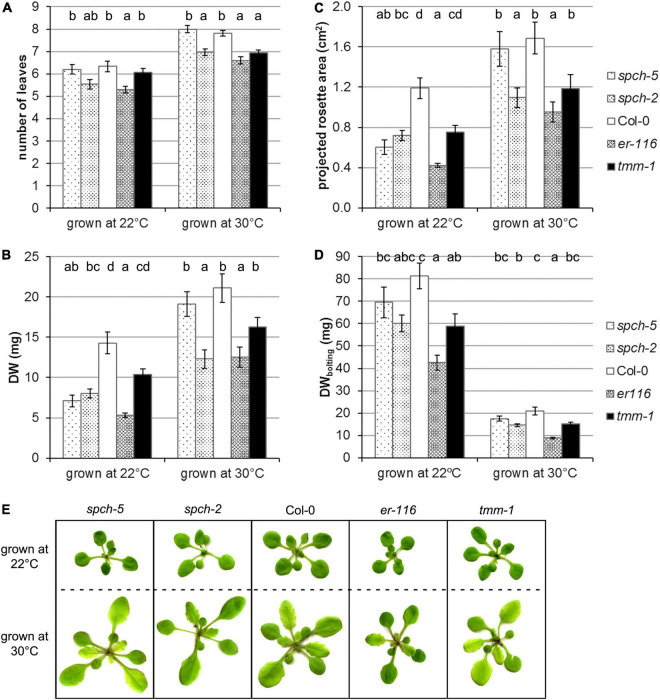
Supra-optimal temperature effects on the growth of selected genotypes. Plant growth was evaluated in terms of **(A)** number of leaves in the rosette, **(B)** dry weight, and **(C)** the projected plant area at 15 dag. **(D)** Dry weight at bolting (24 dag for 22°C and 18 dag for 30°C). **(E)** RGB images of plants of the same age (15 dag) grown at optimal conditions (22°C) or at supra-optimal temperature (30°C). Values are means ± SE (*n* = 20). Different letters above the bars indicate statistically significant differences between genotypes at the same growth temperature (*p* ≤ 0.05) by one-way ANOVA followed by Dunnet’s T3 (22°C data in panels **B,C**; 30°C in panel **D**) or the Tukey’s test (22°C data in panel **D**), or by the Kruskal–Wallis test followed by the Dunn’s test (22°C data in panel **A**; 30°C data in panels **A**–**C**).

Growth was assessed for these genotypes in two temperature conditions (22 and 30°C) by measuring number of leaves, projected the rosette area and DW in the plants of 15 dag (Figures 3A–C,E). As described (reviewed by Casal and Balasubramanian, 2019), supra-optimal temperature accelerated development and induced morphological changes in Col-0. All the mutants examined showed wild-type responses to warm conditions. At 22°C, Col-0 had produced 6.3 leaves, while, at 30°C, the plants had 7.8 leaves (Figure 3A). This behavior was statistically equal at both temperatures in *spch-5*, which formed 6.2 leaves at optimal temperature and 8 at supra-optimal temperature. However, the rest of the genotypes produced a lower number of leaves than Col-0 or *spch-5* when grown at 30°C, yet only *er-116* had fewer leaves at 22°C.

Interestingly, *spch-5*, even though smaller than Col-0 as measured by the projected rosette area and DW when grown at 22 and at 30°C, developed the same DW (Figure 3B) and the same projected plant area (Figure 3C) at 15 dag than the wild type. In contrast, *spch-2* and *er-116* were generally smaller than Col-0 under both temperature regimes, measured as number of leaves, the projected rosette area, and DW (Figures 3A–C,E). The *tmm-1* mutant only showed a statistically significant decrease in the number of leaves when grown at 30°C.

Since all genotypes accelerated their growth at supra-optimal temperature and shortened their vegetative phase by 1 week at the expense of biomass accumulation, at the time of bolting, the plants grown at 22°C (at 24 dag) had produced about 400% more biomass than the same genotype grown at 30°C (at 18 dag). It is worth noticing that the *spch-5* mutant showed the closest DW values to those of Col-0 at bolting time, regardless of the growing conditions (Figure 3D).

Figure 3E shows representative plants of the same age (15 dag) grown under optimal or supra-optimal temperature. At 30°C, all the genotypes tested displayed the canonical thermomorphogenic phenotypes: they produced more leaves (Figure 3A), with elongated petioles and ellipsoid laminas, and all of them flowered about a week earlier than at 22°C. However, morphological differences among mutants were appreciated, as for *spch-2* having leaves more elongated than the rest of genotypes and *er-116*, showing more compact rosettes and rounder leaves. In contrast, *spch-5* and *tmm-1* showed no changes in their morphology relative to the wild type.

#### Effects of supra-optimal temperature on anatomical leaf traits

**FIGURE 4:**
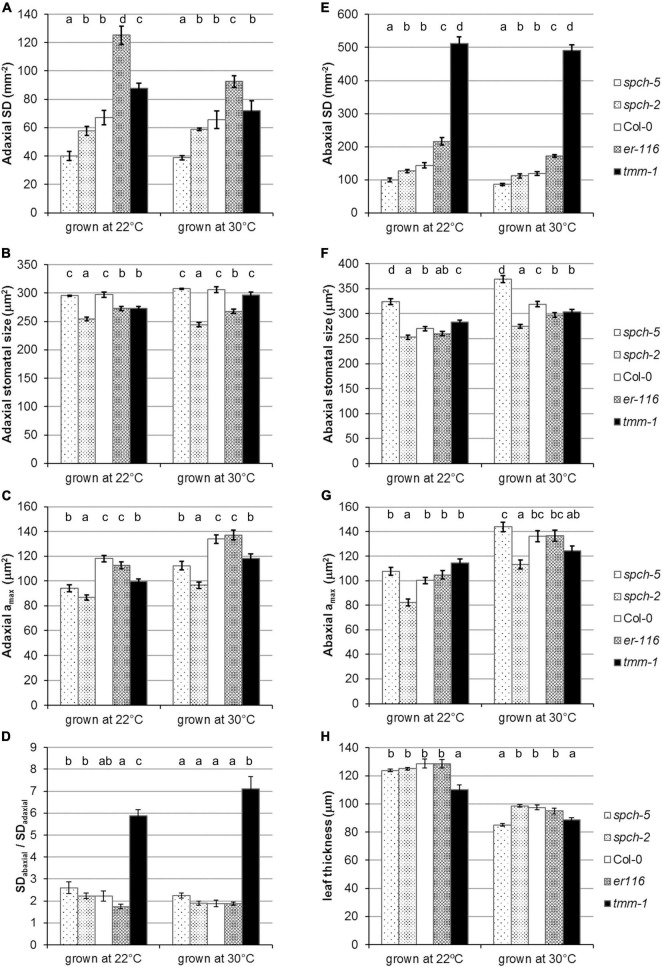
Stomatal phenotypes under optimal and supra-optimal growth temperature. Stomatal density **(A,E)** and stomatal size **(B,F)** for adaxial and abaxial side, respectively, of fully expanded leaves. **(C,G)** Maximum stomatal pore size (*a*_max_) on the adaxial and abaxial sides of the leaf, respectively, at 23 dag for 22°C and 17 dag for 30°C. **(D)** Stomatal density ratio SD_*abaxial*_/SD_*adaxial*_. **(H)** Thickness of mature leaves from plants grown under 22 and 30°C. Values are means ± SE, calculated from *n* = 7 **(A–G)** and *n* = 6–18 **(H)**. Different letters above the bars indicate statistically significant differences between genotypes at the same growth temperature (*p* ≤ 0.05) according to one-way ANOVA followed by the Dunnet’s T3 test (30°C data in panel **A**) or the Kruskal–Wallis test followed by the Dunn’s test (the rest of data).

Since growth at supra-optimal temperatures reduces stomata production in Arabidopsis (Crawford et al., 2012), we examined SD in the adaxial and abaxial epidermis of fully expanded leaves of the different genotypes grown at 22 and at 30°C (Figures 4A,E). As expected, the abaxial SD of Col-0 decreased by an 18% at 30°C and all the mutants followed the same trend (*t*-test *p* < 0.05), except for *tmm-1*. Compared to Col-0, the decreases were lower in *spch* mutants (14–15%), slightly larger in *er-116* (21%), and marginal in *tmm-1* (4%). In the adaxial side, only high stomatal abundance genotypes showed a decrease in SD at 30°C (*t*-test *p* < 0.05), being more pronounced in *er-116*. Although SD changes were more prominent in the abaxial side, the ratio of stomata on the abaxial and adaxial leaf surfaces (SD abaxial/SD adaxial) in all genotypes was similar in both growth conditions (Figure 4D). Stomatal size differed among genotypes and leaf sides at both growth temperatures (Figures 4B,F). Col-0 and *spch-5* had adaxial stomata of equal size, which tend to be slightly larger at 30°C. Abaxial stomata were, however, much larger in *spch-5* than in any other genotype at 22°C and, particularly, at 30°C. Despite its very low SD, the larger abaxial stomata of *spch-5* translated into an *a*_max_ that equals that of Col-0 at both temperatures. More notably, *spch-5 a*_max_ also equals (the adaxial side) or is even larger (the abaxial side) than the *a*_max_ of the high-SD mutant *tmm-1* when grown at 30°C. In contrast, *spch-2* had the smallest stomata and *a*_max_ values in both epidermis and conditions, in spite of having a higher SD than *spch-5* (Figures 4C,G). These data suggest that *spch-5* might compensate its extremely low SD with larger abaxial stomata that provide a larger pore area for gas exchange, and that these traits might be particularly important for physiological performance at supra-optimal temperature.

As expected from previous findings (Poorter et al., 2016), the leaves developed under elevated temperature were thinner than those formed at optimal temperature for all the genotypes when examined at bolting time (Figure 4H). This adaptive response to supra-optimal temperature was particularly strong in *spch-5*, whose leaves were much thinner than those of Col-0 and most of other lines examined. The leaf structure of *spch-5* was further inspected with respect to that of Col-0 (Supplementary Figure 3), quantifying the cross-sectional area occupied by the spongy and palisade mesophyll layers, and by the upper and lower epidermal surfaces (Supplementary Figure 3A). This analysis revealed leaf anatomical differences among *spch-5* and Col-0 at both growth conditions, mainly due to a distinct contribution of the mesophyll tissue types (Supplementary Figure 3B). While at 22°C, the Col-0 palisade and spongy mesophylls had similar thickness (54 and 58 μm, respectively), the palisade tissue predominated in the *spch-5* leaf (64 vs. 37 μm). Growth at supra-optimal temperature decreased the palisade layer in both genotypes but reduced the spongy tissue only in Col-0 (*t*-test *p* = 0.002), resulting in mesophylls with thicker spongy than palisade layers in Col-0 (49 and 29 μm, respectively) but of nearly identical thickness in *spch-5* (33 μm for spongy and 32 μm of palisade). It is worth noting that the palisade layer (the primary photosynthetic tissue) of *spch-5* was, compared to Col-0, thicker at 22°C and equal at 30°C (*t*-test *p* = 0.0009 and *p* = 0.29, respectively). Interestingly, while supra-optimal temperature also caused an increase in the thickness of the adaxial epidermis in both genotypes (*t*-test *p* < 0.00001), the abaxial epidermis became thinner only in *spch-5* leaves (*t*-test *p* = 0.0003).

**FIGURE 5:**
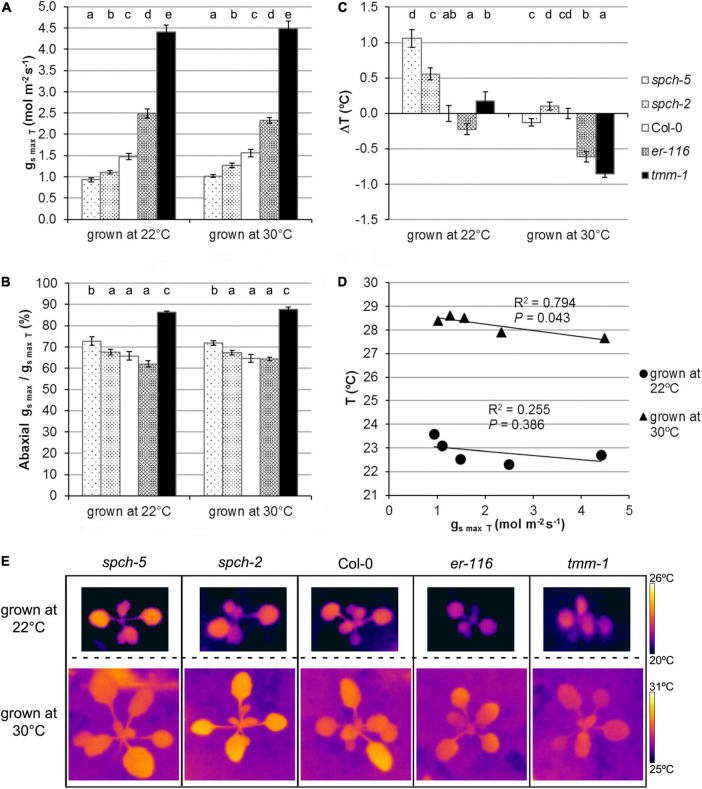
Stomatal conductance under optimal and supra-optimal growth temperature. **(A)** Calculated total *g*_smax_ for the leaf, **(B)** the fraction contributed by the abaxial *g*_smax_ for fully expanded leaves, and **(C)** alteration in leaf temperature relative to the wild type. Values are means ± SE, calculated from *n* = 7 **(A,B)** and *n* = 20 **(C)**. Different letters above the bars indicate statistically significant differences between genotypes at the same growth temperature (*p* ≤ 0.05) according to the Kruskal–Wallis test followed by the Dunn’s test. **(D)** Relationship between leaf temperature and the total *g*_smax_. The mean values of each genotype at a given growth temperature were used for the regression analysis. **(E)** Thermographic images of whole plants at 15 dag.

The maximum potential for stomatal conductance (*g*_smax_; see Section “Materials and methods”) can be calculated from stomatal anatomical traits. To estimate the theoretical potential for transpiration, we calculated *g*_smax_ for the adaxial and abaxial sides and the total *g*_smax_(*g*_smaxT_) at the two growth temperatures in fully expanded leaves of our genotype panel (Figure 5A). Not surprisingly, those with higher SD had higher *g*_smaxT_ than Col-0, while the opposite was found for the *spch* mutants, with all genotypes behaving similarly at both growth temperatures. However, *spch-5*, even having the same ratio of abaxial to adaxial SD than Col-0 (Figure 4D), showed a higher contribution of the abaxial *g*_smax_ to the *g*_smaxT_ in both growth conditions (Figure 5B). Although *g*_smax_ correlates well with operating (measured) stomatal conductance (Franks et al., 2009; Dow et al., 2014), we recorded the leaf temperature as an experimental estimation of transpiration in our conditions (Scarth et al., 1948; Milthorpe and Spencer, 1957). Figures 5C,E show the temperature change of the different genotypes relative to Col-0, recorded for optimum and supra-optimum growth temperature. While, at 22°C, the *spch* mutants were 0.6–1.1°C warmer than Col-0, at supra-optimal temperature, they were indistinguishable from Col-0. The high SD mutants had the opposite behavior: similar to Col-0 at 22°C but cooler when grown at 30°C (Figure 5D). Therefore, both *spch* mutants would transpire at a rate similar to Col-0 when exposed to supra-optimal temperature but sustained a lower transpiration when grown at optimal temperature. Thus, leaf temperature and *g*_smaxT_ showed a significant and positive correlation for plants grown at 30°C but not at 22°C. The apparent discrepancy for the low SD genotypes between calculated *g*_*s*maxT_ values, which were much lower than the Col-0 value, and leaf temperature, statistically similar for the two *spch* mutants and Col-0 at supra-optimal temperature, should then relate to differences in additional traits, such as leaf thickness and rosette morphology.

#### Impact of supra-optimal growth temperature on photosynthetic activity and secondary metabolism

**FIGURE 6:**
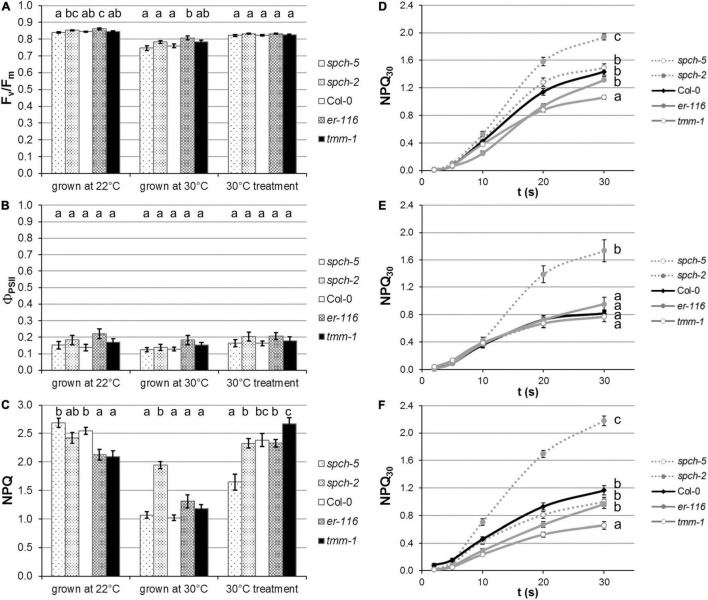
Impact of temperature regime on plant performance. Photosynthetic activity is evaluated in terms of **(A)** maximal efficiency of PSII (F_*V*_/F_*M*_), as well as **(B)** quantum yield of PSII in steady state (Φ_PSII_). **(C)** The capacity for energy dissipation in the chloroplast is analyzed by the non-photochemical quenching in steady state (NPQ). Kinetics of NPQ activation in plants grown at **(D)** optimal conditions (22°C), **(E)** supra-optimal temperature (30°C), and **(F)** after a short-term treatment at 30°C. Values are means ± SE (*n* = 20). Different letters above the bars indicate statistically significant differences between genotypes at the same growth temperature (*p* ≤ 0.05) according to one-way ANOVA followed by the Dunnet’s T3 test (30°C treatment data in panel **A**), the Tukey’s test **(F)**, or the Kruskal–Wallis test followed by the Dunn’s test (the rest of data).

Growth at supra-optimal temperature caused a slight inhibition of photosynthesis, regardless of the genotype, as shown by the lower Fv/Fm values relative to the plants grown at optimal temperature (Figure 6A). However, this minor permanent inhibition of PSII did not have an impact on the electron transport rate of the thylakoid, since Φ_PSII_ was not affected significantly (Figure 6B). Plants under stress often increase their capacity for energy dissipation in the thylakoid as a mechanism of acclimation to challenging environmental conditions. In plants grown at 30°C, and taking into account the moderate decrease of their Fv/Fm values, the energy dissipation mechanisms (measured by NPQ; Figure 6C) were less active than at 22°C, suggesting that the supra-optimal temperature did not impose a photosynthetic stress in all the genotypes, except for *spch-2*. To gain a better insight into the capacity for energy dissipation at PSII, we analyzed the kinetics of NPQ activation (related to the de-epoxidation state of the xanthophyll cycle, Figures 6D,E). Compared to Col-0, this activation was markedly faster for *spch-2*, particularly at 30°C, and slower for *tmm-1*, while no significant differences were found between *er-116*, *spch-5*, and Col-0.

To test to what extent these differential responses related to anatomical traits acquired during plant development at both temperatures, a short-term acclimation treatment of plants grown at 22°C was carried out by exposing them to 30°C 3 days prior to measurements. After this treatment, *spch-5* seemed to respond better than the rest of genotypes. Relative to Col-0, no significant changes in Fv/Fm (Figure 6A) or Φ_PSII_ (Figure 6B) were found for any mutant. However, *spch-5* had lower NPQ values, suggestive of a better short-term acclimation to the new environment than the other four genotypes (Figure 6C). Moreover, the kinetics of NPQ activation (Figure 6F) showed that short-term treatment did not significantly alter the rate of activation of energy dissipation mechanisms underlying NPQ in any mutant compared to when they were grown at 22°C. Under any treatment, *spch-2* showed a significantly faster activation kinetics than the rest of genotypes (Figure 6F), indicating that *spch-2* has a more limited capacity for acclimation to elevated temperature.

To further estimate plant fitness, several VIs were obtained by hyperspectral reflectance imaging (Supplementary Figure 4). These indexes (see Section “Materials and methods” for details) are related to growth and physiology and are indicative of plant vigor and photosynthetic activity (GNDVI, PRI), stress (RVSI), pigments (PSSR2), content on chlorophylls (CARI), anthocyanins (ANTH2), and carotenes (CAR2), and leaf structure (mRENVDI).

A green normalized difference vegetation index values indicated that chlorophylls were less abundant in plants grown at supra-optimal temperature (Supplementary Figure 4A). Similarly, the PRI index pointed toward a decrease in the photosynthetic activity (Supplementary Figure 4B), a trend was also observed in the measured Φ_PSII_ (Figure 6B). According to RVSI and mRENDVI, all genotypes would be experiencing a mild stress when cultivated at the supra-optimal temperature, being the mutants with high SD the most affected by the growing condition (Supplementary Figures 4C,H). The *spch* mutants showed a singular profile with respect to Col-0, specially for the VIs that estimate photosystem pigments, suggesting that these mutants have a higher capacity for light absorption (the CARI index; Supplementary Figure 4E) and anthocyanin accumulation (ANTH2; Supplementary Figure 4F) when grown at supra-optimal temperature, while also showed the altered chl*a*/*b* ratio and carotenoid content at optimal temperature (PSSR2 and CAR2; Supplementary Figures 4D,G).

### Altered stomatal density affects adaptability to supra-optimal temperature growth

**FIGURE 7:**
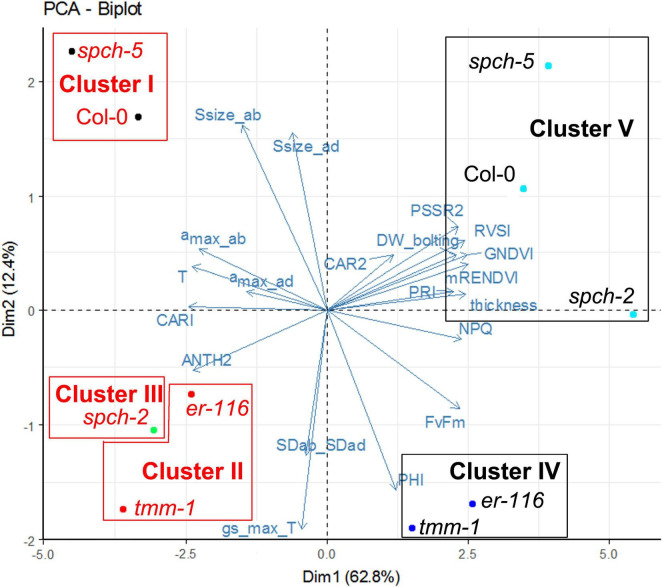
Clustering of selected genotypes according to their phenotypes grown under optimal and supra-optimal temperature. The Biplot showing the first two variables and the individual factor map of the PCA. Dim1 and Dim2 are first and second principal components, respectively. Their percentage of explained variance is given in parentheses. Genotype color indicates the growth temperature, 22°C (black) or 30°C (red).

To further describe the characteristics of growth at supra-optimal temperature, a PCA followed by a hierarchical clustering was performed on a selection of anatomical, physiological, and VI traits (Figure 7 and Supplementary Figure 5). The first two principal components explained 75% of the variance among genotypes and growth conditions (Supplementary Figure 5A). Stomatal size on the abaxial side of the leaf (and the adaxial side to a smaller extent) and Φ_PSII_ (as the activity rate of PSII) had a strong positive contribution to PC1, whereas the *g*_smaxT_ and the ratio SD_*ab*_/SD_*ad*_, to a lesser extent, had a strong negative contribution to PC1. For PC2, DW_*bolting*_, leaf thickness, and the inhibition of PSII (F_*V*_/F_*M*_ and NPQ), together with the VIs related to leaf structure (mRENVDI), plant vigor and stress (GNDVI, PRI, RVSI), and pigments (PSSR2) had a strong positive contribution. The *a*_max_ in the abaxial side of the leaf and leaf temperature (as an indirect measurement of stomatal conductance), together with VIs related to pigment contents (CARI and ANTH2), had a strong negative contribution (Supplementary Figure 5B).

The phenotypes shown by the five genotypes grown at optimal and supra-optimal temperature were analyzed by HCPC. The optimal number of clusters was found by cutting the generated tree, following the inertia gain criterion. Thus, genotypes were grouped in five clusters, represented in Figure 7. Growth temperature caused stronger effects on phenotype than the mutations under study, since genotypes were first grouped according to their growing conditions (in Clusters IV and V for 22°C vs. Clusters I, II, and III for 30°C). Among the genotypes grown at 22°C, Cluster IV was formed by the mutants with high SD (*er-116* and *tmm-1*), and Cluster V consisted of Col-0 and the low SD *spch* mutants. Genotypes grown under 30°C clustered similarly with the exception of *spch-2*, which had its own cluster III, closer to high SD mutants instead of clustering with Col-0 and *spch-5*. It is remarkable that *spch-5*, with distinct anatomical differences as compared to Col-0 (much lower SD and leaf thickness and higher stomatal size) grouped together with the wild type, and both genotypes behaved very similarly when grown at supra-optimal temperature.

## Discussion

Stomatal abundance and distribution determine the maximum potential for gas exchange, impinging on key physiological and performance parameters, such as photosynthesis, cooling capacity, water use efficiency or biomass accumulation, both in Arabidopsis and crop species (Dow et al., 2014; Bertolino et al., 2019; Buckley et al., 2020). Our initial panel of high- and low-SD mutants grown at optimal temperature showed mild alterations in photosynthetic parameters. These parameters were not always directly related to the severity of their epidermal phenotypes. Several studies comparing the effects on plant fitness of mutations in different genes, leading to lower or higher SD, had varied outcomes (von Groll et al., 2002; Doheny-Adams et al., 2012; Franks et al., 2015; Hepworth et al., 2015; Dow et al., 2017; Morales-Navarro et al., 2018; Caine et al., 2019; Dunn et al., 2019). In a survey of 62 wild Arabidopsis accessions, the correlation between cotyledon and leaf SD values was positive and significant (*r* = 0.7; Delgado et al., 2011). In our experiments, cotyledons and leaves in most genotypes showed a similar behavior but also showed differences that might have effects for seedling establishment (cotyledons) and plant performance (leaves). All the mutants with an increase in SD, either moderate (*epf1-1, epf2-3*, *er-116*, and *er-105*) or severe (*sdd1-3* and *tmm-1*), showed various alterations of PSII behavior (Figures 2A–G). The low-SD mutant *spch-2* appeared less affected, although characteristically showed a fast activation kinetics of the photoprotection mechanisms (Supplementary Figure 2), suggestive of a chronic mild stress. Remarkably, *spch-5* photosynthesis was as efficient as in Col-0, despite the very low SD of this mutant. Contrasting with these results, the effect on photosynthesis was found concordant to the reduction in SD caused by the manipulation or mutation of other regulatory genes. For example, the yield of wheat lines with severely low SD caused by the overexpression of *EPF1* was lower than other lines with a moderate decrease in SD (Hepworth et al., 2015; Dunn et al., 2019). Transpiration, estimated by cotyledon and leaf temperature, confirmed that, as expected, the two low-SD genotypes sustained lower water losses than the wild type in both organs, while higher-SD genotypes had generally higher transpiration in leaves and some of them also in cotyledons (Figures 2D,H). These differences in cooling capacity suggested that the mutants might respond differently to elevated temperature.

To explore this assumption, a subset of the mutants with no or mild alterations in photosynthetic parameters was grown at supra-optimal temperature. All genotypes underwent developmental changes indicative of a typical thermomorphogenic response, such as accelerated growth, elongated petioles, and early flowering (Figure 3; Casal and Balasubramanian, 2019). These results indicate that the mutations that altered stomatal abundance did not substantially affect the capacity of the plants to adapt their growth to elevated temperature. This finding is relevant, since genotype-specific differences in the developmental responses to growth temperature have been reported for a panel of natural Arabidopsis accessions (Ibañez et al., 2017). Supra-optimal temperature slightly exceeded the adaptation capacity of the photosynthetic machinery for all genotypes, including the wild type, as shown by the slight decrease in their F_*V*_/F_*M*_ values (Figure 6A). However, only *spch-2* appeared to be stressed (Figure 6C). Moreover, among the different mutants examined, *spch-5* showed a notable behavior at 30°C, being its number of leaves and rosette area indistinguishable from those exhibited by the wild type. The same was true for the biomass accumulated at 15 dag by both genotypes at supra-optimal, but not at optimal temperature, as, at 22°C, the *spch-5* plants were much smaller than Col-0 (Figures 3B,C), in accordance with its reported slower development (de Marcos et al., 2017). However, *spch-5* plants grown at 22°C reached the same DW values than Col-0 at bolting time.

In Col-0, thermomorphogenesis includes changes in anatomical leaf traits, notably a lower SD in the abaxial epidermis (Crawford et al., 2012). In our growing conditions, all genotypes showed this abaxial change, except for *tmm*-*1*. We found that, while Col-0 and low-SD mutants maintained the adaxial SD unchanged at supra-optimal temperature, high-SD mutants also decreased SD in the adaxial side (Figure 4). This could be due to the fact that adaxial stomatal formation takes place very early during leaf primordia development (Geisler and Sack, 2002). In our experimental setting, the seedlings were at this time still growing at 22°C prior to their exposure to supra-optimal temperature (to prevent deleterious effects of high temperature on germination and early seedling development), while, in the abaxial epidermis, stomatal development extends for much longer, and, thence, it takes place when our plants were already exposed to supra-optimal temperature. Because of this, we cannot exclude that growth at 30°C since leaf initiation might lead to even lower adaxial (and probably abaxial) stomatal densities. By contrast to this and other studies in cotyledons (Figure 1; MacAlister et al., 2007; de Marcos et al., 2017), we found no significant differences in SD between *spch-2* and Col-0 for both leaf epidermis. The variation in phenotype severity across different organs and epidermal sides is commonly observed in stomatal mutants and might reflect specific regulatory contexts for stomatal development (see, for example, Geisler et al., 1998; Dow et al., 2014). These regulatory differences may also underlie the greater SD decreases in adaxial than in the abaxial side observed for *er-116* and *tmm*-*1* leaves at supra-optimal temperature.

With the exception of *tmm-1*, the mutants showed the same abaxial: the adaxial SD ratio than Col-0 (Figure 4D). Differing from previous reports (Crawford et al., 2012), we found that, in the abaxial epidermis, where the SD decreases were more pronounced, stomatal size increased significantly at supra-optimal temperature (Figure 4). This fits with the described compensation effect between cell size and number in leaves (Hisanaga et al., 2015), which also applies to stomatal size and abundance and that has been interpreted as a means to maintain the transpiration potential when stomata numbers decrease (Doheny-Adams et al., 2012; Dittberner et al., 2018; Buckley et al., 2020). Interestingly, although stomatal size and density show a strong negative correlation among *A. thaliana* accessions, only stomata size positively covaries with water-use efficiency, suggesting that stomatal size directly impacts on the trade-off between photosynthesis and transpiration (Dittberner et al., 2018). In the context of the stomatal abundance decrease triggered by elevated temperatures, our findings suggest that stomatal size compensation might improve cooling capacity at supra-optimal temperatures.

The notable similarities of *spch-5* and Col-0 plants in morphology and growth at elevated temperature (Figure 3) concur with profound differences in anatomical leaf traits between the two genotypes. Adaxial and abaxial SDs were lower and abaxial stomatal size much larger in *spch-5* than in Col-0 (Figures 4A,E,F). Thus, the full compensation between cell number and size described for this mutant at optimal temperature (de Marcos et al., 2017) is maintained in plants grown at 30°C. At both growth temperatures, changes in stomatal size were more prominent in the abaxial side, resulting in a maximum stomatal pore area (*a*_max_) equal in *spch-5* than in Col-0 in this leaf side, while the adaxial value was lower in the mutant (Figure 5). Using these anatomical parameters to calculate the theoretical maximum stomatal conductance (*g*_smaxT_), we found that *spch-5* overall values were lower, although its potential abaxial conductance was significantly higher than in Col-0, and despite having the same abaxial:adaxial SD ratio. Thus, although at supra-optimal temperature, *spch-5* stomata experienced significant anatomical changes in comparison to Col-0, they did not translate into a better theoretical cooling capacity. However, indirect determination of transpiration showed that, at supra-optimal temperature, the mutant holds values indistinguishable from the wild type, what was surprising because, at optimal temperature, *spch-5* was a warm, low transpiration mutant. This unexpected finding might be explained by the decrease in leaf thickness elicited by growth at supra-optimal temperature, which was observed in all genotypes as previously reported for Col-0 (Crawford et al., 2012; Vile et al., 2012) but resulted remarkably significant in *spch-5*. A decrease in leaf thickness, a thermomorphogenic response, would contribute to a decrease of the CO_2_ diffusion path length and thus to an increase in mesophyll conductance (Vile et al., 2012), perhaps allowing *spch-5* to keep higher carbon fixation and growth rates, comparable or even higher than for Col-0. Moreover, the short-term acclimation to elevated temperature of *spch-5* was remarkably robust, as suggested by the NPQ activation rate, comparable to that of Col-0 (Figures 6D–F). In turn, this finding is indicative of a similar short-term acclimation capacity of *spch-5* and Col-0, despite the low SD of this mutant, which was expected to be a constraint to photosynthesis. All these features could explain why the *spch-5* mutation does not cause a decrease in plant size or biomass relative to the wild type at bolting time (Figure 3). In contrast, supra-optimal temperatures exceeded the adaptation capacity of *spch-2* chloroplasts to ambient conditions, as shown by its faster NPQ activation kinetics.

In *spch-5*, leaf structure was also impacted by supra-optimal T, with a higher proportion of the spongy versus palisade mesophyll compared to plants grown at 22°C, what might favor gas diffusion and thus leaf conductance to CO_2_ and H_2_O and, therefore, transpiration. In addition to these effects, the improved *spch-5* cooling capacity at 30°C could be due to changes in stomatal dynamics, as it has been recently established that elevated temperatures promote stomata opening (Kostaki et al., 2020).

There is clear evidence for a developmental coordination between epidermal and inner leaf tissues (Savaldi-Goldstein et al., 2007) and, more specifically, between stomata and mesophyll (reviewed by Baillie and Fleming, 2020). For instance, the stomata-promoting peptide STOMAGEN is produced in the mesophyll and determines stomata formation in the epidermis (Kondo et al., 2010), and mesophyll air spaces as substomatal cavities only form associated with stomata (Serna and Fenoll, 2000; Bergmann, 2004; Lundgren et al., 2019), but the mechanisms controlling this developmental effect are unknown. Interestingly, a hyperactive form of SPCH (SPCH-24-A) that increased stomata production led to thicker leaves (Dow et al., 2017), while the reduced SD of the hypomorphic *spch-5* promotes the opposite effect (this work). Dow et al. (2017) also reported that manipulation of other stomatal development genes translated into changes in mesophyll cell size and density, but, again, the molecular signals underlying such effects remain unknown. More recently, Lundgren et al. (2019) have reported that, in wheat and in Arabidopsis, mesophyll airspaces and stomata distribution are tightly linked and that this link depends on the presence of functional stomata sustaining gas exchange, and not simply stomatal precursors or guard cells. Thus, the available information indicates that both developmentally programmed signaling between stomatal lineages and mesophyll, and physiological cues linked to stomata functioning act together to shape inner leaf tissues, depending on stomatal abundance and patterning. This coordinated development is crucial to establish specific transpiration and photosynthesis activities and, thence, water use efficiency.

The developmental and functional effects of growth at supra-optimal temperature are supported by the results of PCA with a selection of anatomical, physiological, and VIs traits, as the hierarchical clustering classified the phenotypes first according to the temperature of growth (Figure 7). Within each growth temperature, high-SD genotypes grouped together, not surprisingly. Low-SD genotypes behaved, however, differently at optimal (at which both clustered with Col-0) and supra-optimal temperature, where, while *spch-5* grouped with the wild type, *spch-2* had its own cluster very close to that of the high-SD mutants. The main traits explaining the variance among genotypes were stomatal size (mostly abaxial but also adaxial), the activity rate of photosystem II (Φ_PSII_), theoretical maximal conductance (*g*_smaxT_), and the SD ratio between abaxial and adaxial sides for the first dimension. In the second dimension, the main traits were biomass at bolting time (DW_*bolting*_), leaf thickness, and several traits related to inhibition of photosynthesis, plant vigor, and stress, as well as the abaxial maximal pore size (*a*_max_) and leaf temperature. The close clustering of *spch-5* and Col-0, particularly at 30°C, was surprising, given their significant anatomical differences and the important contribution of anatomical traits to our PCA and cluster construction. It is worth noticing that growth at 30°C results in *spch-2*, reaching lower values than Col-0 in number of leaves, the projected rosette area, DW and DW at bolting (Figure 3). Regarding stomatal traits (Figure 4), *spch-2* is only similar to Col-0 in SD but shows lower stomatal size and lower *a*_max_ in both epidermis. This concurs with a faster NPQ induction (Figure 6), particularly at 30°C, indicative of a limited capacity of this mutant for acclimation to elevated temperature. Indirect effects of the *spch-2* mutation, i.e., on leaf development could alter mesophyll anatomy and, therefore, water and CO_2_ conductance and photosynthesis (as reviewed by Flexas et al., 2016), and might, in part, underlie this behavior. Thus, we found striking differences between *spch-5* and *spch-2* for relevant physiological, growth, and anatomical parameters and for their response to supra-optimal temperature. Their molecular lesions affect separate SPCH domains, the DNA-binding domain in *spch-5*, and the C-terminal of yet uncharacterized function in *spch-2* (MacAlister et al., 2007; de Marcos et al., 2017). How these molecular lesions affect the regulatory network in which SPCH participates is unknown, but they likely stem from allele-specific regulatory dysfunctions.

Our results show that the anatomical and morphological responses to supra-optimal are genotype specific and not simply a consequence of SD. The behavior of the very low-SD mutant *spch-5* in comparison with Col-0 further indicates that characteristics essential for fitness at supra-optimal temperature, such as cooling capacity and biomass accumulation, can be achieved through diverse anatomical solutions. *spch-5* exemplifies how genotype-specific differential thermo-morphogenetic responses convert a low SD, warm, and slow-developing mutant into a successful genotype that performed similar to a wild-type plant under non-favorable conditions.

## Data availability statement

The raw data supporting the conclusions of this article will be made available by the authors, without undue reservation.

## Author contributions

MP-B and MM designed the experiments and analyzed the data with contributions of CF and MB. MP-B performed physiological, growth, and leaf thickness analysis. JI-M, AM-F, and AM determined the stomatal phenotypes. MM, MP-B, and CF wrote the manuscript with inputs from MB. All authors read and approved the final manuscript.

## Conflict of interest

The authors declare that the research was conducted in the absence of any commercial or financial relationships that could be construed as a potential conflict of interest.

## Publisher’s note

All claims expressed in this article are solely those of the authors and do not necessarily represent those of their affiliated organizations, or those of the publisher, the editors and the reviewers. Any product that may be evaluated in this article, or claim that may be made by its manufacturer, is not guaranteed or endorsed by the publisher.

## Supplementary material

Supplementary Figure 1 Stomatal density in the abaxial cotyledon epidermis of Col-0 and stomata developmental mutants. Values are means ± SE (*n* = 10), obtained by counts from fully expanded cotyledons (23 dag). Incidence of single, clustered, and total stomata is depicted. Different letters above the bars indicate statistically significant differences between genotypes (*p* ≤ 0.05) according to one-way ANOVA followed by the Dunnet’s T3 test (isolated and total stomata data) or the Kruskal–Wallis test followed by the Dunn’s test (clustered stomata data).

Supplementary Figure 2 **(A,B)** Kinetics of non-photochemical quenching (NPQ) activation in cotyledons and **(C,D)** in fully expanded leaves of the indicated genotypes. Values are means ± SE (*n* = 20). Asterisks indicate significant differences to wild-type Col-0 (one-way ANOVA followed by the two-sided Dunnett’s test). *, ^**^, and ^***^ correspond to *p* < 0.05, 0.01, and 0.001, respectively.

Supplementary Figure 3 **(A)** Micrographs of leaf cross-sections of mutants grown under optimal and supra-optimal temperature. **(B)** Measured thickness of palisade and spongy mesophyll, adaxial, and abaxial epidermis of Col-0 and *spch-5* plants grown at 22 or 30°C. Values are means ± SE (*n* = 13–18). ^***^ correspond to *p* < 0.001 according to a Student’s *t*-test.

Supplementary Figure 4 Hyperspectral vegetation indexes related to physiology (**A**, GNDVI) and (**B**, PRI); stress (**C**, RVSI); pigments (**D**, PSSR2); content on chlorophylls (**E**, CARI), anthocyanins (**F**, ANTH2), and carotenes (**G**, CAR2); and structure (**H**, mRENVDI) on plants grown under 22 or 30°C. Values are means ± SE (*n* = 20). Asterisks indicate significant differences to wild-type Col-0 (one-way ANOVA followed by the two-sided Dunnett’s test) at the same growth temperature. *, ^**^, and ^***^ correspond to *p* < 0.05, 0.01, and 0.001, respectively.

Supplementary Figure 5 Principal components analysis (PCA) analysis of physiological and anatomical parameters, and vegetation indices. **(A)** Eigen values, percentage of explained variance, and cumulative explained variance for the principal components. Panels **B,C** show the contribution and the cos^2^ value, respectively, of each parameter for each principal component.
