# Supplementary material for: An extremely low stomatal density mutant overcomes cooling limitations at supra-optimal temperature by adjusting stomatal size and leaf thickness
Source: Front Plant Sci. 2022 Jul 22;13:919299. doi: 10.3389/fpls.2022.919299 (PMC9355609; doi:10.3389/fpls.2022.919299)
Supplement: Supplementary file 1 [file Data_Sheet_1.PDF]

Supplementary Table 1. Parameter symbols and descriptions.

Symbol	Description
a_{max}	Maximum stomatal pore size in the adaxial (adaxial a_{max}; a_{max_ad}) or abaxial (abaxial a_{max}; a_{max_ab}) side of the leaf
ANTH2	Anthocyanin reflectance index
CAR2	Carotenoid reflectance index
CARI	Chlorophyll absorption reflectance index
Ci	Internal concentration of CO2
DW	Dry weight of rosette prior to bolting
$DW_{bolting}$	Dry weight of rosette at bolting time
F_0	Minimum chlorophyll fluorescence in the dark-adapted state
Fm	Maximum chlorophyll fluorescence in the dark-adapted state
Fm’	Maximum chlorophyll fluorescence in the light-adapted state
Ft	Chlorophyll fluorescence in the light-adapted state
Fv	Variable fluorescence (Fm – F_0)
Φ_{PSII}	Effective quantum efficiency of PSII
GNDVI	Green normalized difference vegetation index
g_{smax}	Maximum stomatal conductance to water vapor in the adaxial (adaxial g_{smax}) or abaxial (adaxial g_{smax}) side of the leaf
g_{smaxT}	Total maximum stomatal conductance to water vapor (adaxial g_{smax} + abaxial g_{smax})
HCPC	Hierarchical clustering on principal components
mRENVDI	Modified red edge normalized difference vegetation index
NPQ	Non-photochemical quenching
PRI	Photochemical reflectance index
PSSR2	Pigment specific spectral ratio
RVSI	Red-edge vegetation stress index
SD	Stomatal density in the adaxial ($SD_{adaxial}$; SD_{ad}) or abaxial ($SD_{abaxial}$; SD_{ab}) side of the leaf
S_{size}	Stomatal size in the adaxial (S_{size_ad}) or abaxial (S_{size_ab}) side of the leaf
