# Supplementary figures and images for: An extremely low stomatal density mutant overcomes cooling limitations at supra-optimal temperature by adjusting stomatal size and leaf thickness
Source: Front Plant Sci. 2022 Jul 22;13:919299. doi: 10.3389/fpls.2022.919299 (PMC9355609; doi:10.3389/fpls.2022.919299)

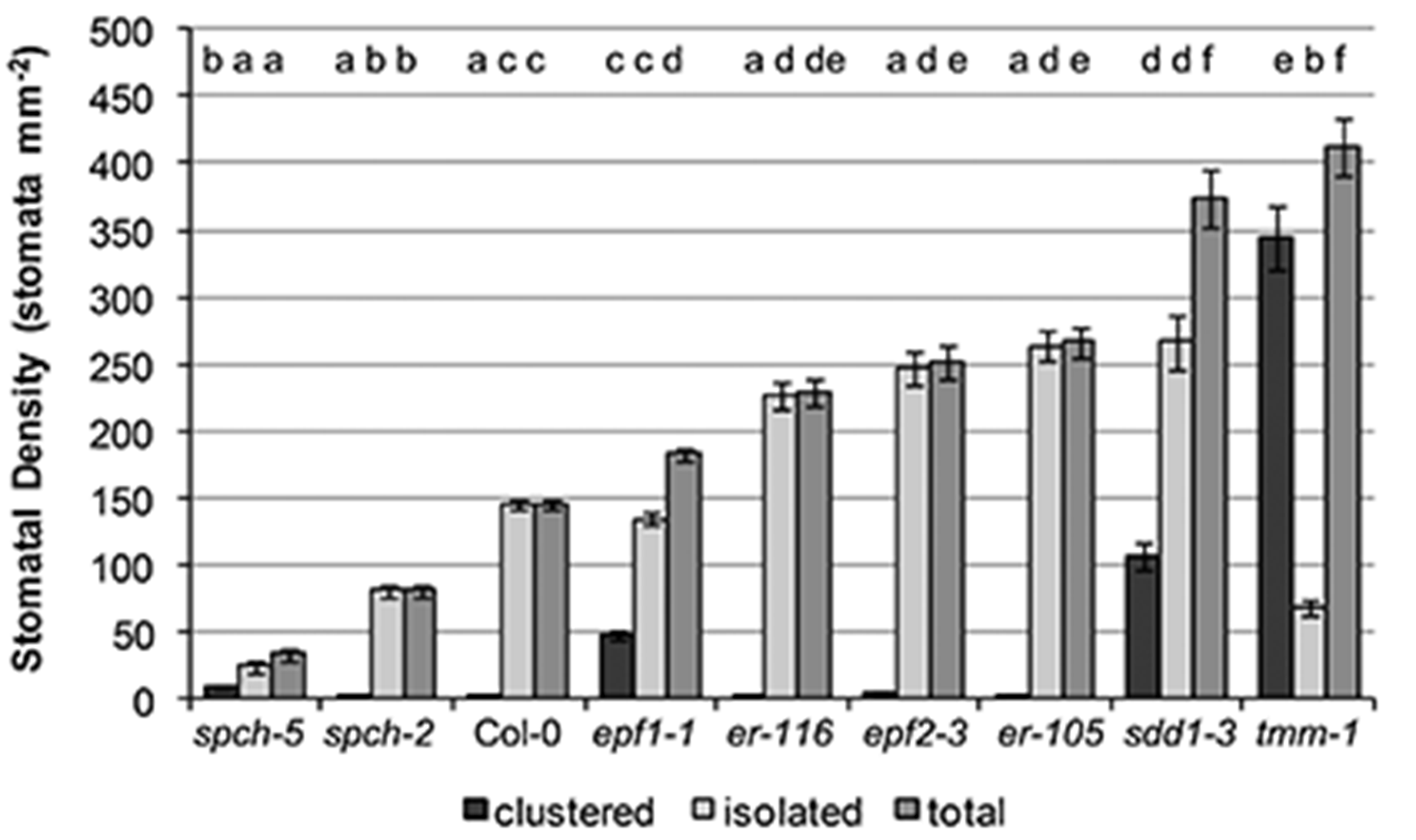

Supplement: Supplementary Figure 1 — Stomatal density in the abaxial cotyledon epidermis of Col-0 and stomata developmental mutants. Values are means ± SE (n = 10), obtained by counts from fully expanded cotyledons (23 dag). Incidence of single, clustered, and total stomata is depicted. Different letters above the bars indicate statistically significant differences between genotypes (p ≤ 0.05) according to one-way ANOVA followed by the Dunnet’s T3 test (isolated and total stomata data) or the Kruskal–Wallis test followed by the Dunn’s test (clustered stomata data). [file Image_1.TIF]

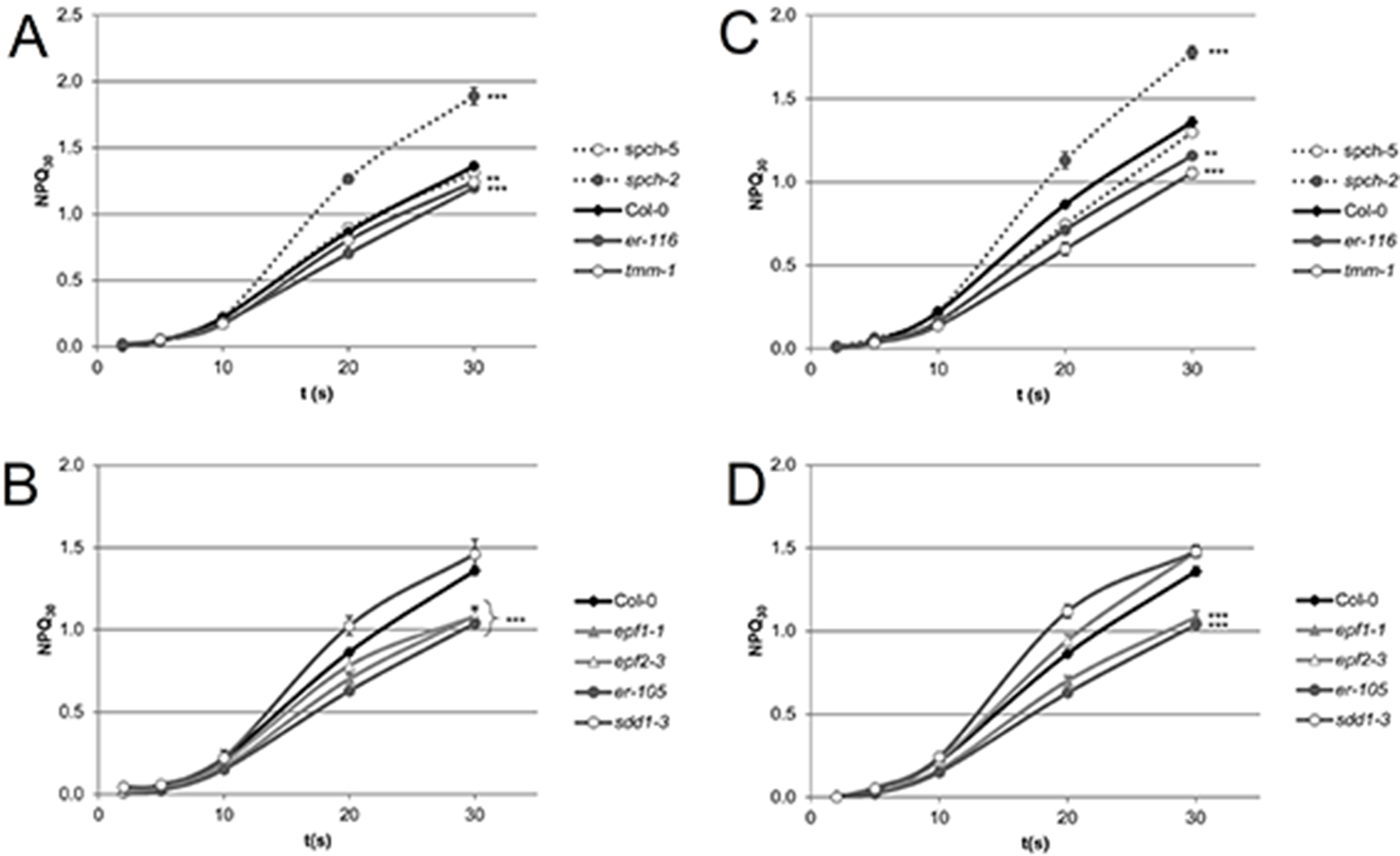

Supplement: Supplementary Figure 2 — (A,B) Kinetics of non-photochemical quenching (NPQ) activation in cotyledons and (C,D) in fully expanded leaves of the indicated genotypes. Values are means ± SE (n = 20). Asterisks indicate significant differences to wild-type Col-0 (one-way ANOVA followed by the two-sided Dunnett’s test). *, **, and *** correspond to p < 0.05, 0.01, and 0.001, respectively. [file Image_2.TIFF]

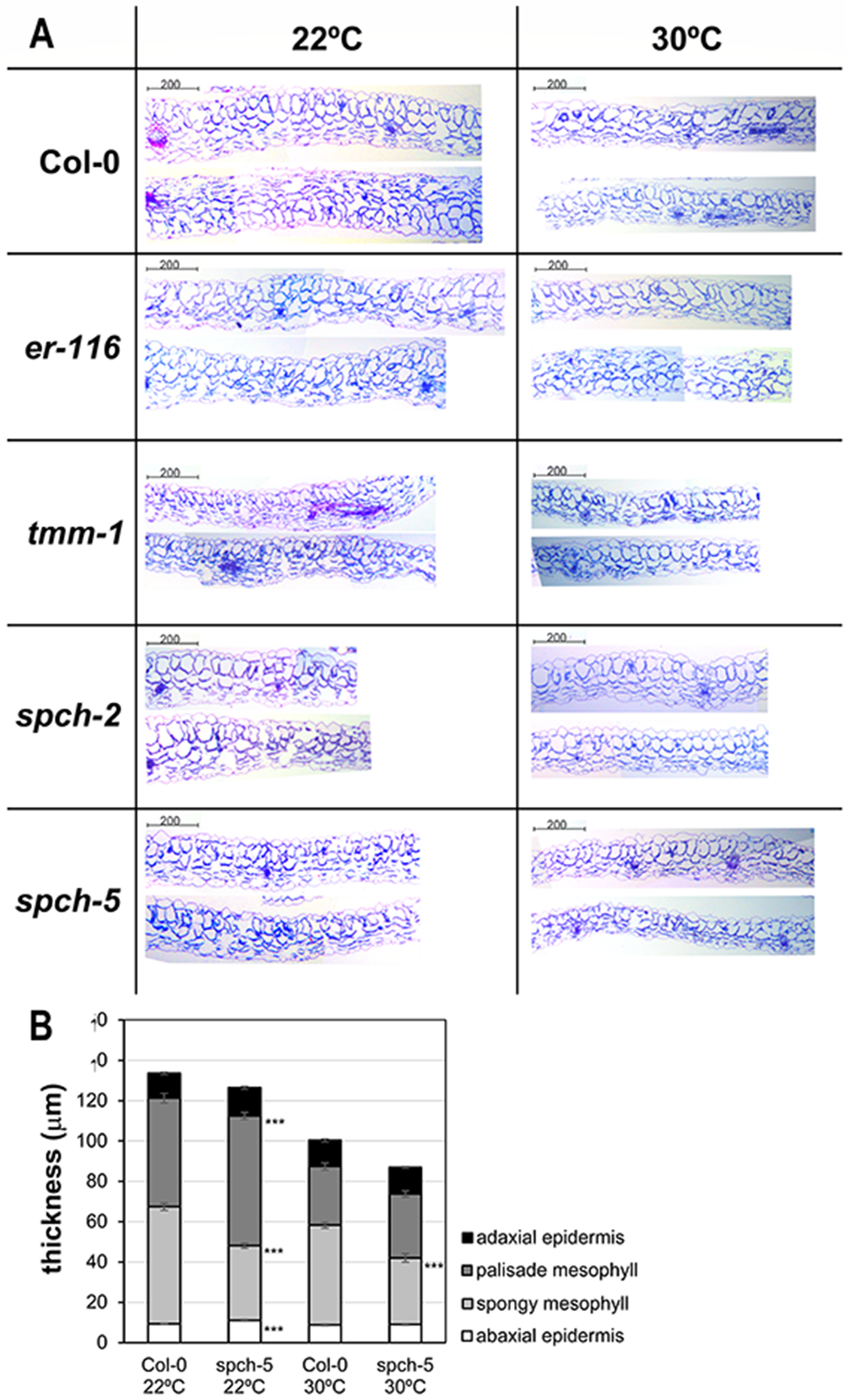

Supplement: Supplementary Figure 3 — (A) Micrographs of leaf cross-sections of mutants grown under optimal and supra-optimal temperature. (B) Measured thickness of palisade and spongy mesophyll, adaxial, and abaxial epidermis of Col-0 and spch-5 plants grown at 22 or 30°C. Values are means ± SE (n = 13–18). *** correspond to p < 0.001 according to a Student’s t-test. [file Image_3.TIFF]

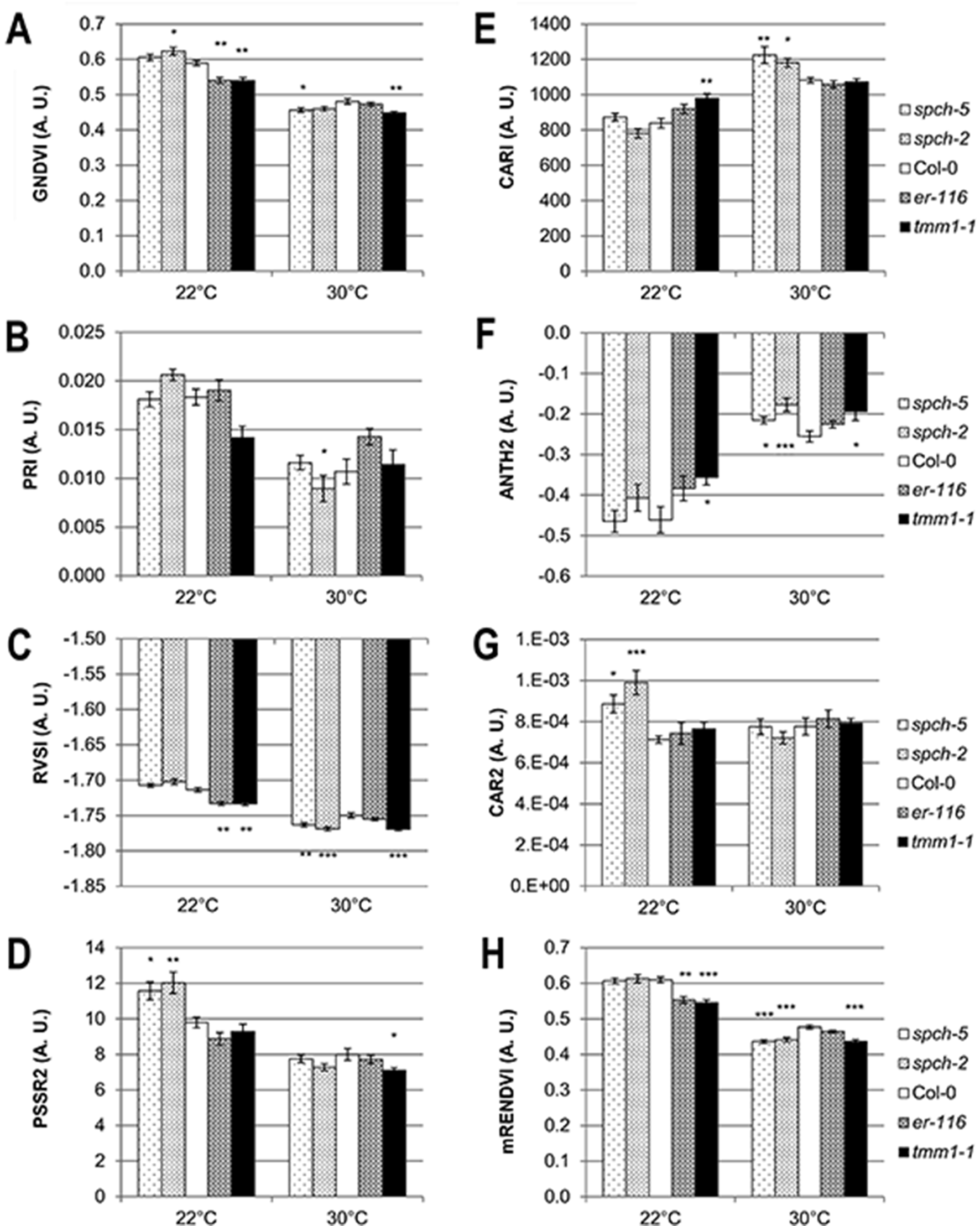

Supplement: Supplementary Figure 4 — Hyperspectral vegetation indexes related to physiology (A, GNDVI) and (B, PRI); stress (C, RVSI); pigments (D, PSSR2); content on chlorophylls (E, CARI), anthocyanins (F, ANTH2), and carotenes (G, CAR2); and structure (H, mRENVDI) on plants grown under 22 or 30°C. Values are means ± SE (n = 20). Asterisks indicate significant differences to wild-type Col-0 (one-way ANOVA followed by the two-sided Dunnett’s test) at the same growth temperature. *, **, and *** correspond to p < 0.05, 0.01, and 0.001, respectively. [file Image_4.TIFF]

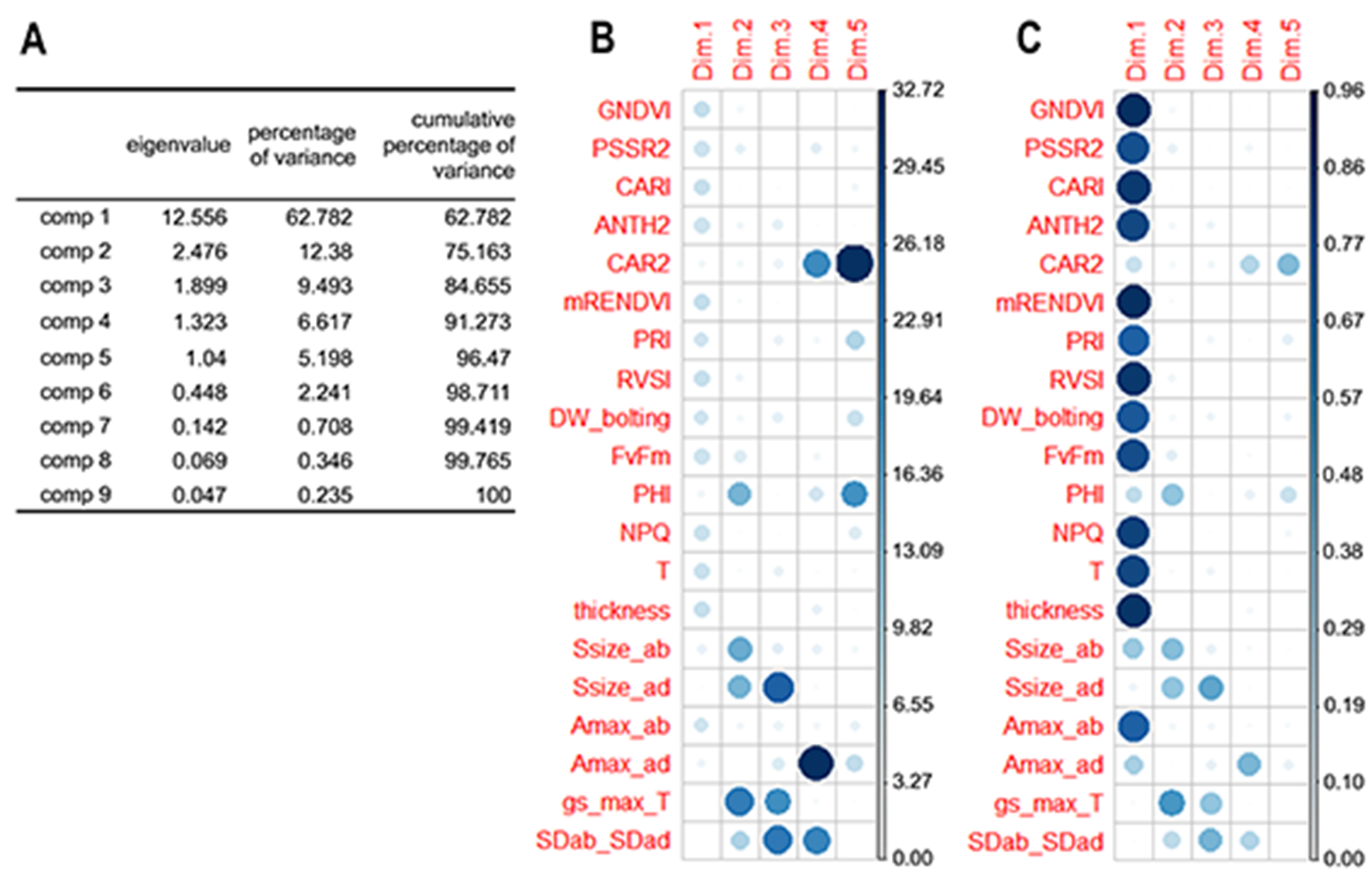

Supplement: Supplementary Figure 5 — Principal components analysis (PCA) analysis of physiological and anatomical parameters, and vegetation indices. (A) Eigen values, percentage of explained variance, and cumulative explained variance for the principal components. Panels B,C show the contribution and the cos2 value, respectively, of each parameter for each principal component. [file Image_5.TIFF]
